# Effect of a new type of head and neck fixation and traction device in the surgical treatment of patients with irreducible atlantoaxial dislocation

**DOI:** 10.1186/s12893-023-01957-0

**Published:** 2023-03-30

**Authors:** Haiyi He, Xiang Li, Peng Li, Kai Zhang, Pengfei Zhang, Qiang Guo, Cheng Dong, Gaosheng Guo, Fuxiang Nie, Juan Du

**Affiliations:** 1 Sanmenxia Central Hospital, Henan University of Science and Technology, 472000 Sanmenxia, China; 2 Department of Rehabilitation, Shiyan Renmin Hospital, 442099 Shiyan, China

**Keywords:** Fixation traction device, Irreducible atlantoaxial dislocation, Clinical operation, Quality of life

## Abstract

**Background:**

In order to improve the clinical medical technology, enhance the clinical effect and improve the disease detection rate, the clinical incidence rate of atlantoaxial dislocation and vertebral body malformation that are difficult to summarize is increasing year by year.

**Methods:**

A total of 80 patients with atlantoaxial dislocation deformity treated in our hospital from January 2017 to May 2021 are selected for this study. According to the number table method, 80 patients are randomly divided into the auxiliary group and the traditional group, with 40 cases in each group. The traditional group is treated with posterior atlantoaxial pedicle screw system internal fixation and intervertebral fusion, and the auxiliary implementation and application of a new head and neck fixation and traction device through nasal cannula and oral release decompression fixation for posterior fusion. The patients in the two groups are compared changes and differences in efficacy, spinal cord function index, pain score, surgery, and quality of life.

**Results:**

Compared with the traditional group, the total clinical effective rate, cervical spine extension and flexion range of motion, physical function, physical function, psychological function, and social function in the auxiliary group are significantly improved. The operation time, intraoperative blood loss, and VAS score are significantly reduced (P < 0.05).

**Conclusion:**

The new head and neck fixation traction device can improve the surgical efficacy and quality of life of patients with irreversible atlantoaxial dislocation, enhance spinal cord function, reduce pain symptoms and surgical risks, and is worthy of clinical application.

## Background

In order to improve clinical medical technology, enhance clinical effects, and improve disease detection rate, the clinical incidence of atlantoaxial dislocation and vertebral body deformity, which is difficult to summarize, is increasing year by year. There are many causes and complex pathological mechanisms, mainly including congenital Deformity, inflammatory reaction and severe trauma lead to a sharp increase in the difficulty of clinical treatment, which seriously affects the treatment effect and prognosis of patients [1]. At present, posterior decompression and reduction and occipitocervical fusion and fixation are mostly adopted as clinical treatment schemes, but the efficacy of some patients still fails to meet expectations, which may cause cervical and occipitocervical injuries in severe cases [2].

Nasal intubation through oral release decompression reduction and posterior fusion fixation therapy is a better solution for non-surgical posterior decompression reduction therapy combined with internal fixation therapy. The auxiliary application of traction devices can effectively fix, thereby enhancing the fixation effect [3]. The main contributions of this paper are as follows:

1. The effect of a new head and neck fixed traction device in the surgical treatment of irreducible atlantoaxial dislocation is analyzed in this paper.
2. The posterior fusion treatment for patients with atlantoaxial dislocation and new head and neck malformation, supplemented by nasal intubation, oral decompression and fixation, and fixed traction device, can improve the fixation effect, help to improve the surgical effect of patients, and improve the quality of life of patients.

The rest of this paper is organized as follows: Sect. 2 is the general Information data and evaluation criteria. The comparison of surgical index and changes of spinal cord function are discussed in Sect. 3. Section 4 discusses this paper. Section 5 concludes the paper with summary.

## Methods

### General information

A total of 80 patients with irretrievable atlantoaxial dislocation who are treated in our hospital from January 2017 to May 2021 are selected for investigation and research. 80 patients are randomly included in the auxiliary group and traditional group according to the number table method, with 40 cases in each group. In the auxiliary group, the male to female ratio is 30/10, the age ranged from 27 to 57 years, with an average of (44.32 ± 10.95) years. According to the ASIA standard classification, 12 cases are grade C, 15 cases are grade D, and 13 cases are grade E. In the traditional group, the ratio of male to female is 28/12, the age is 26–58 years old, and the mean age is (43.28 ± 11.21) years old. There are 13 cases of grade C, 14 cases of grade D and 13 cases of grade E, respectively(*P* > 0.05) and comparable. All procedures in this study are consistent with the ethical principles of the Declaration of Helsinki. For the implementation of the nasal cannula oral release stress reduction and fixation posterior fusion treatment without surgery contraindications and severe allergic reaction, the object of study and their families are in details about research significance. The presence of infectious diseases, malignant tumors, congenital atlantoaxial dislocation, cognitive disorders, motor neuron diseases, psychiatric disorders, and any of the other trials in the same period should be excluded from the study.

### Experimental methods

In the traditional group, the posterior arch 18-20 mm outside the midpoint of the posterior atlantoaxial tubercle is selected as the approach point of the atlas pedicle. The approach point is the intersection of the central vertical line of the lateral mass at the lower edge of the posterior atlantoaxial arch and the upper 2 mm waterline. Keep the vertical entry direction of the nail on the horizontal plane, and ensure that the upward cutting angle of the nail on the sagittal plane is 6 °. The axial pedicle screw insertion point is located at the middle point of the upper quadrant in the back of the lower axial joint. The direction of nail insertion is 10–15 ° internal angle and 20–30 ° upper angle.

**Fig. 1:**
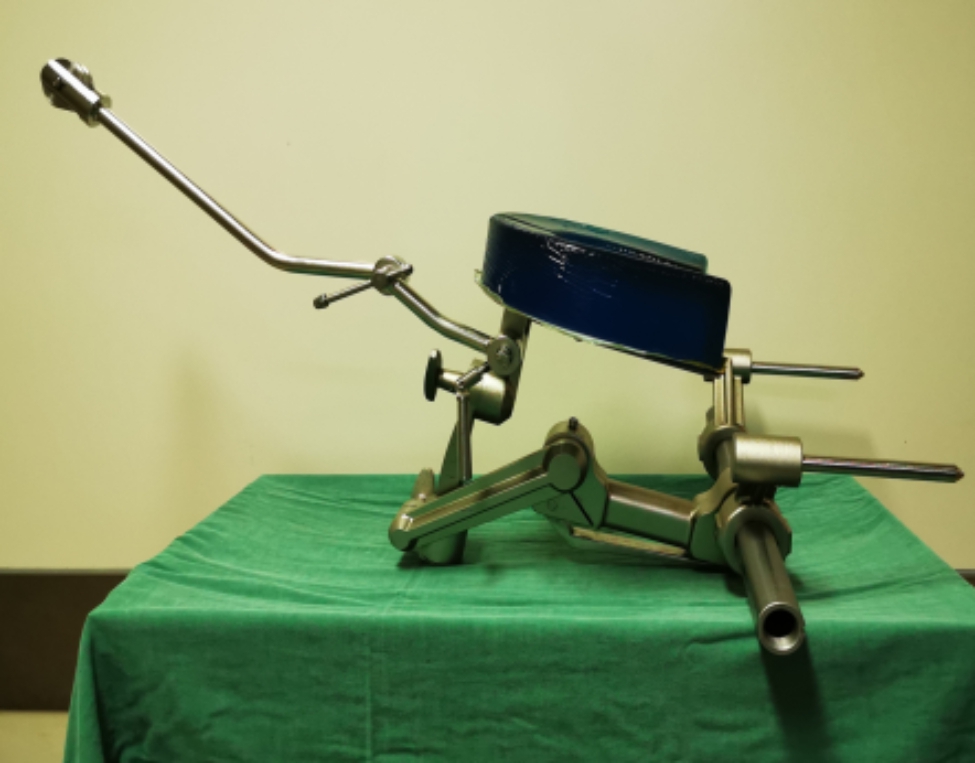
New head and neck fixation traction device

The auxiliary group is treated with nasal intubation oral release decompression reduction and posterior fusion fixation assisted with the new head and neck fixation traction device: (1) Preoperative preparation. The patient is gargling with compound chlorhexidine 3 times a day, 5 min/ time, 1 week before surgery. Check the patient’s oral health and hygiene, including infection lesions, upper and lower incisors stability, dentures, etc. Check the patient’s nasal stenosis, if the above conditions, use tracheotomy. Broad-spectrum antibiotics is given intravenously 30 min before operation, and no respiratory tract infection is confirmed by examination. (2) Surgical plan. General anesthesia through nasal intubation, traction of cervical vertebra in supine position, new head and neck fixed traction device used for 2 cm amphibians to maintain traction status, gradually increasing from 3 to 15 kg below body weight. C-arm X-ray machine confirmed that it was an irreversible atlantoaxial joint dislocation fluoroscopy technology. Regularly disinfect, lay towels and install mouth plugs, pour weak iodophor water into the mouth and soak it for 5 min. Apply light drops. After repeated rinsing with physiological salt iodine, place a red urine tube into the mouth, pull the suture bundle of the tongue back along the edge, so that the posterior pharyngeal wall is fully exposed. The atlantoaxial lateral mass joint is closely adhered. The weight of hyperextension traction was increased to 12 kg in case of 1/5 − 1/3 fibrous hyperplasia and hyperosteogeny under the anterior arch of atlantoaxial vertebrae. After the adhesion joint was opened, the anterior and inferior motion of the axial odontoid process was observed by c-arm x-ray fluoroscopy. Figure 1 is the new head and neck fixation traction device. It is clearly evident from Fig. 1 that if the position is well determined by C-arm X-ray machine, the wound is rinsed repeatedly with hydrogen oxygen and normal saline, and the throat wall is sutured with full-thickness suture.

### Evaluation criteria

The clinical efficacy of patients is calculated and the difference is compared. The criteria are as follows: The score improvement rate after treatment is calculated according to the changes of cervical spinal cord function score. The formula is [(post-treatment score - pre-treatment score) /17- pre-treatment score]×100%. The improvement rate of 90-100% is cure, > 60% is significant effect, 25-60% is effective, and < 25% is ineffective. Total effective rate = (cure + significant effect + effective)/total cases ×100%.

VAS is used to evaluate and compare the pain degree of patients at t1-t3 time points. A blank piece of paper marked 0–10 scale is taken, and the subjects are randomly marked on the paper according to their pain degree, and the total score is 0–10. The pain degree of patients increase with the increase of the score.

JOA is used to evaluate cervical spinal cord function, and the total score of JOA ranges from 0 to 17, and dysfunction increases with the decrease of the score.

At T1-T3, the quality of life (psychological function, physiological function, physical function and social function) of patients are scored respectively according to QOLS, and the higher the score, the higher the quality of life of patients.

### Statistical methods

SPSS 26.0 statistical software is used to process the data. The measurement data are expressed as mean ± standard deviation (‾*x* ± *s*) and passed *t* test [4]. Enumeration data are expressed as percentage (%) and passed *x²* test [5]. *F*-test is used for multiple groups of data. The spherical test (Mauchly) is used to compare the data at different time points within the group [6]. *P* > 0.05 indicates that the covariance matrix is full of football symmetry, and *P* < 0.05 indicates that the difference is statistically significant [7].

## Results

### Comparison of postoperative efficacy differences

Table 1 is the difference in total clinical response rate. It is clearly evident from Table 1 that the total clinical response rate in the adjuvant group is significantly higher than that in the conventional group (*P* < 0.05).

**Table 1:** Difference in total clinical response rate (*n* = 40,(%))

Group	Cure	Cure	Effective	No avail	Total effective rate
Auxiliary group	8(20.00)	14(35.00)	14(35.00)	4(10.00)	36(90.00)
Traditional group	6(15.00)	10(25.00)	12(30.00)	12(30.00)	28(70.00)
*x2*					5.000
*P*					0.025

## Comparison of surgical index values

Table 2 is the comparison of numerical differences of surgical indicators. It is clearly evident from Table 2 that the operative time and intraoperative blood loss in the auxiliary group are significantly lower than those in the traditional group (*P* < 0.05).

**Table 2:** Comparison of numerical differences of surgical indicators(*n* = 40,‾*x* ± *s*)

Group	Intraoperative bleeding(ml)	Operation time(h)
Auxiliary group	528.95 ± 22.43	3.21 ± 2.23
Traditional group	619.56 ± 2.74	5.71 ± 2.67
*t*	25.361	4.545
*P*	< 0.001	< 0.001

## Changes in pain scores at different time points

Table 3 is the differences in pain score changes at different time points. It is clearly evident from Table 3 that the VAS scores of 80 subjects show a decreasing trend.

**Table 3:** Differences in pain score changes at different time points(*n* = 40,‾*x* ± *s*)

Group	Time point	VAS score
Auxiliary group	T1	6.55 ± 2.33
	T2	4.60 ± 2.14
	T3	3.23 ± 1.87
Traditional group		
	T1	6.51 ± 2.31
	T2	5.66 ± 2.21
	T3	4.30 ± 1.90
* F* time point		485.878
*P* time point		< 0.001
* F* Time point * group		405.816
*P* Time point * group		< 0.001

**Fig. 2:**
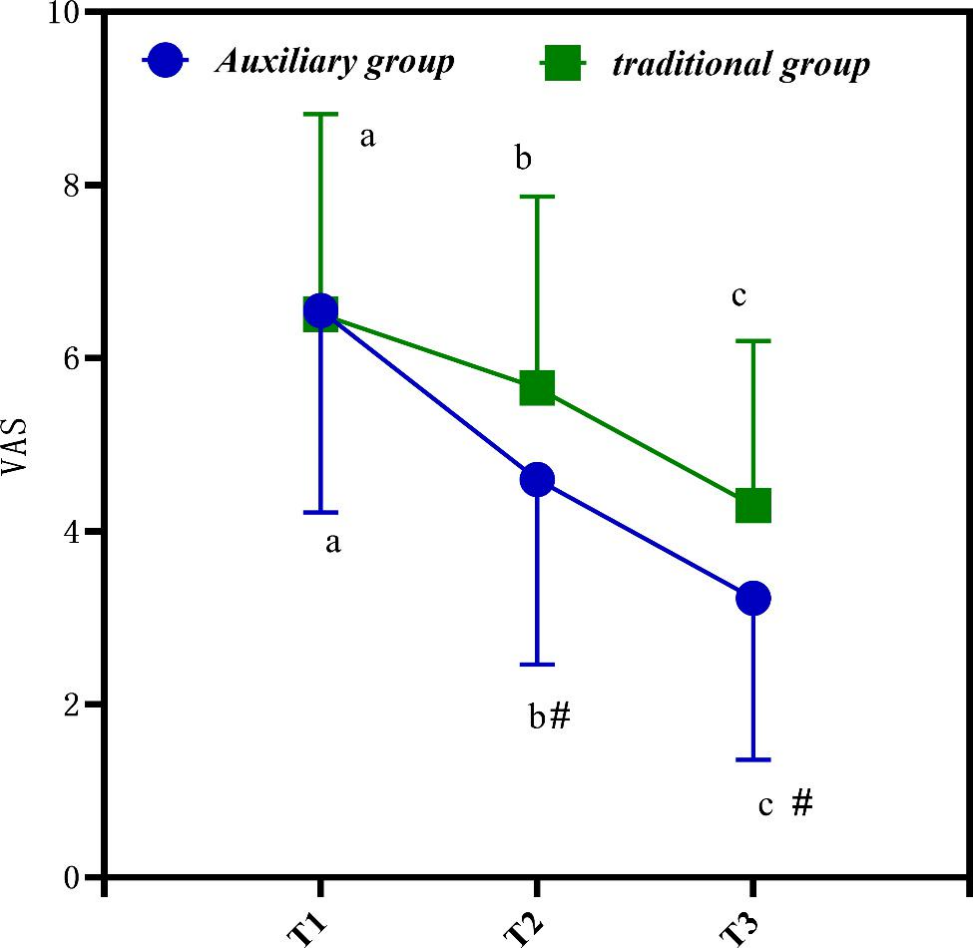
Changes of pain scores at different time points

Figure 2 is the changes of pain scores at different time points. In Fig. 2, a, b, and c represent P < 0.05 compared with other time points. # is compared with traditional group. It is clearly evident from Fig. 2 that the postoperative T2-T3 scores of the auxiliary group are significantly lower, and there are statistical differences in the data (P < 0.05).

## Changes of spinal cord function indexes at different time points

Table 4 is the changes of spinal cord function indexes at different time points. It is clearly evident from Table 4 that the JOA score and cervical extension and flexion range of motion of all subjects show an increasing trend.

**Table 4:** Changes of spinal cord function indexes at different time points (*n* = 40,‾*x* ± *s*)

Group	Time point	JOA score (score)
Auxiliary group	T1	5.60 ± 1.33
	T2	8.85 ± 1.64
	T3	13.60 ± 2.87
Traditional group		
	T1	5.56 ± 2.31
	T2	7.81 ± 2.21
	T3	10.56 ± 1.90
* F* time point		432.433
*P* time point		< 0.001
* F* Time point * group		414.325
*P* Time point * group		< 0.001

**Fig. 3:**
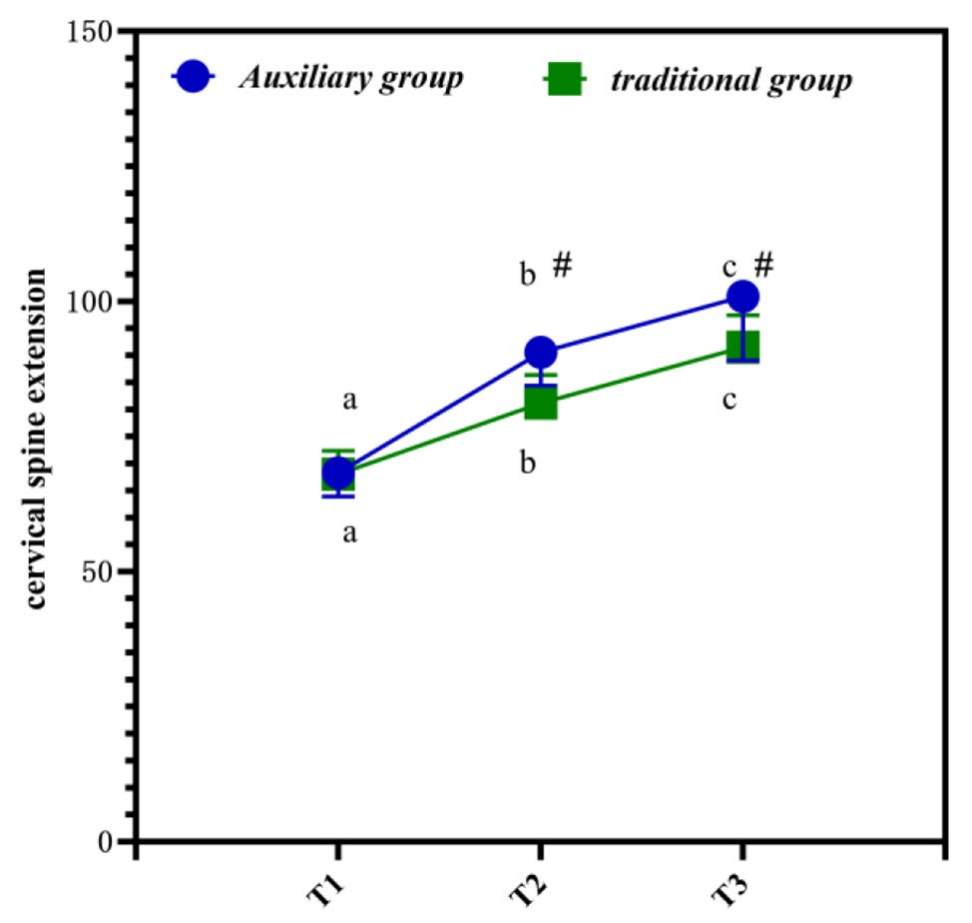
Differences in spinal cord function indicators at different time points

Figure 3 is the differences in spinal cord function indicators at different time points. In Fig. 3, a, b, and c represent P < 0.05 compared with other time points. # is compared with traditional group. It is clearly evident from Fig. 3 that the postoperative T2-T3 index values of the auxiliary group are significantly higher, and the data are statistically different (P < 0.05).

## Changes in QOLS scores at different time points

Table 5 is the changes in QOLS scores at different time points. It is clearly evident from Table 5 that the scores of the four quality of life dimensions of all study subjects increase significantly.

**Table 5:** Changes in QOLS scores at different time points (‾*x* ± *s*)

Group	Time point	Physiologic function	Physical function	Mental function	Social function
Auxiliary group	T1	68.03 ± 10.76	58.78 ± 9.32	53.64 ± 10.65	55.91 ± 7.77
	T2	77.53 ± 10.54	68.28 ± 10.13	60.94 ± 10.45	67.17 ± 7.82
	T3	86.58 ± 10.13	78.50 ± 11.97	71.56 ± 9.89	76.09 ± 7.79
Traditional group					
	T1	68.28 ± 11.12	57.24 ± 9.87	53.92 ± 10.78	55.82 ± 7.56
	T2	73.78 ± 11.10	62.74 ± 11.08	59.42 ± 10.65	61.32 ± 7.56
	T3	78.28 ± 11.09	67.24 ± 12.89	63.92 ± 10.57	66.98 ± 8.23
* F* time point		421.132	414.543	412.623	424.645
*P* time point		< 0.001	< 0.001	< 0.001	< 0.001
* F* Time point * group		523.302	565.377	595.131	563.562
*P* Time point * group		< 0.001	< 0.001	< 0.001	< 0.001

**Fig. 4:**
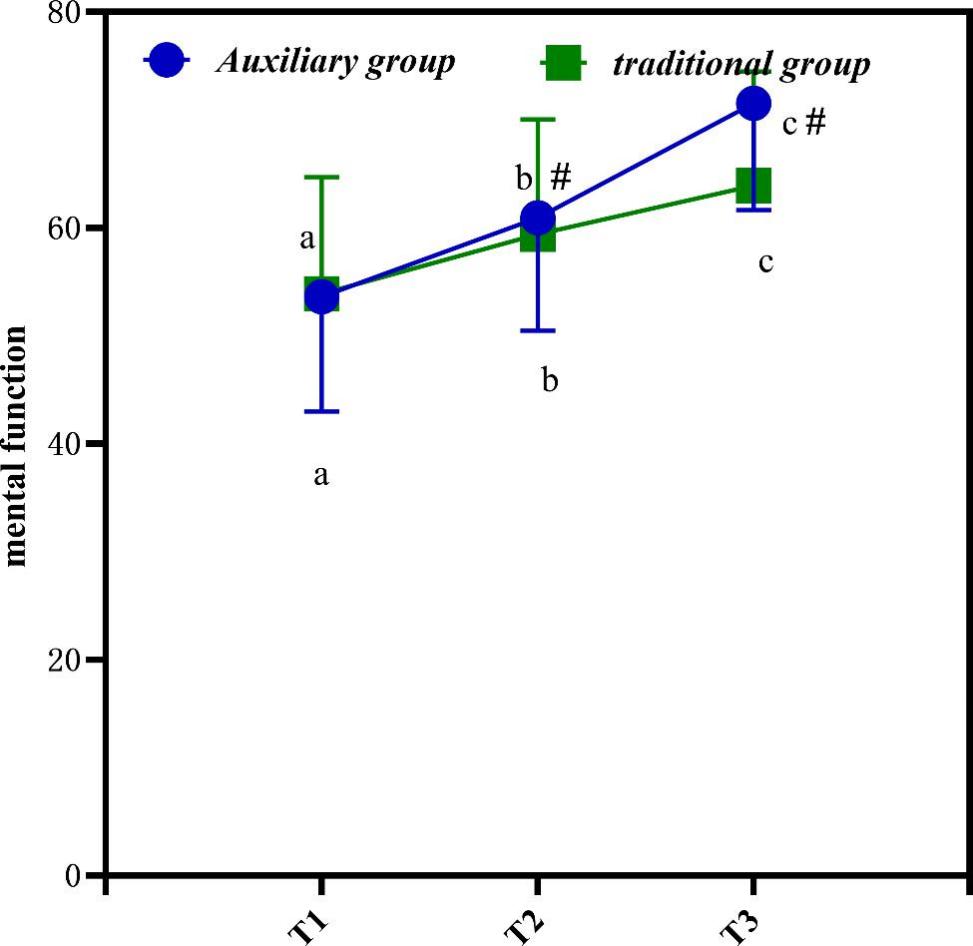
Changes in QOLS scores at different time points

Figure 4 is the changes in QOLS scores at different time points. In Fig. 4, a, b, and c represent P < 0.05 compared with other time points. # is compared with traditional group. It is clearly evident from Fig. 4 that the postoperative T2-T3 index values of the auxiliary group are significantly higher, and the data are statistically different (P < 0.05).

**Fig. 5:**
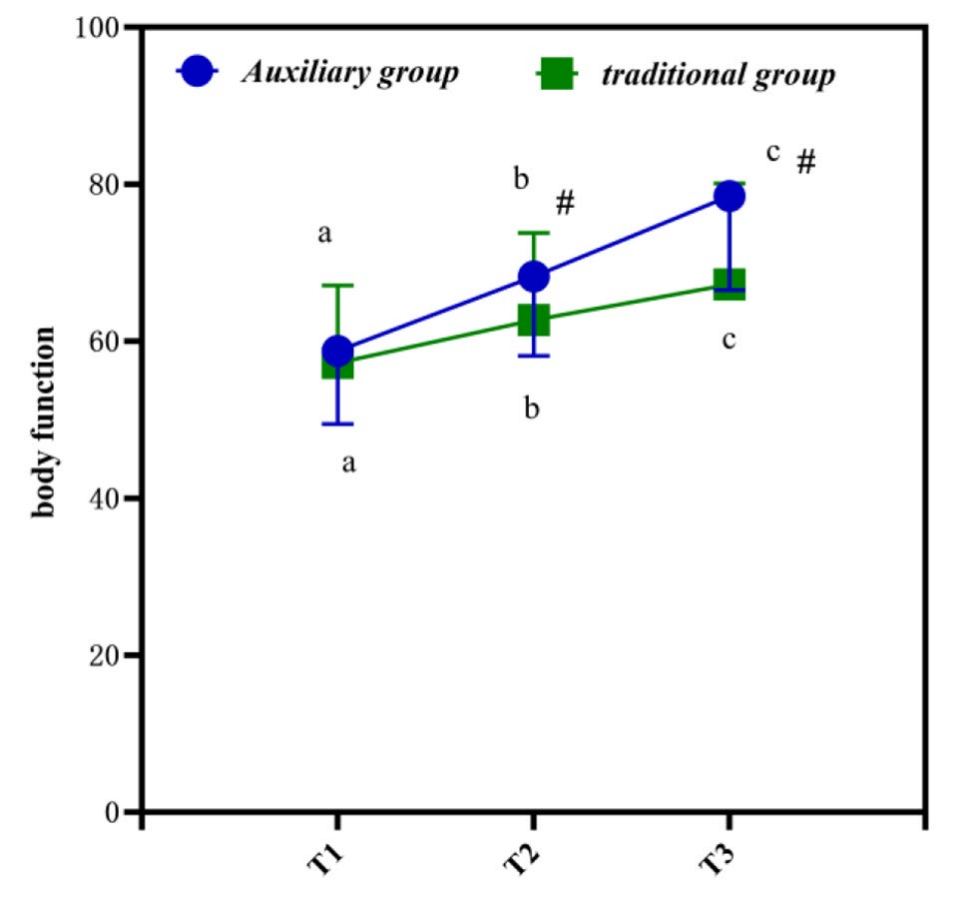
The QOLS score changes at different time points

Figure 5 is the QOLS score changes at different time points. In Fig. 5, a, b, and c represent P < 0.05 compared with other time points. # is compared with traditional group. It is clearly evident from Fig. 5 that the auxiliary group increases more significantly(*P*<0.05).

## Discussion

Atlantoaxial joint dislocation could not be reduced after heavy traction weight was a serious threat to the quality of life of patients. Transoral release, decompression and fixation of nasal cannula was a common solution for the treatment of vertebral dislocation in patients with atlantoaxial abnormality, and its application effect has been confirmed by clinical studies [8]. Nasal catheter is a catheter inserted into the nose for surgery, oxygen delivery, or monitoring purposes. In some cases, the nasogastric tube used to enter the stomach through the nose is also known as the nasal catheter. Without damaging the sinuses or compressing the nasal polyps and other possible growths, it is necessary to carefully place the nasal catheter so that it can pass through the nasal patients, some of whom may need sedatives or local anesthetics when using it. The nasogastric tube passes through the nose and enters the esophagus. The length down to the stomach can vary according to the intended use, and the width is the same.

Traction was also an effective scheme for the treatment of cervical diseases in clinical practice. In addition to correct traction skills, the design of traction device had an important impact on the traction fixation and surgical efficacy of patients [9]. The JOA score of two indexes that could effectively reflect the spinal cord function of patients and the improvement of cervical extension and flexion range of motion were better than those of the traditional group, indicating that the new head and neck fixation device purchased this time could play a positive role in improving the curative effect when the nasal cannula oral released decompression reduction and posterior fusion fixation treatment was applied. However, this operation was difficult and would cause certain injuries to patients, so the auxiliary application of effective traction devices was an important means to improve the efficacy and reduce the trauma [10].

According to the results of this study, the quality of life assisted by the four dimensions of life scored higher. Combined with the results of the analysis, that is, after the posterior fusion and fixation treatment, the implementation of a new head and neck fixed traction device combined with nasal cannula oral release relief reduced the clinical operation time, reduced the risk of bleeding and infection, and then quickly completed the fusion surgery and made it highly stable, which could obtain higher head and neck fixation effect, keep the head of the patient in a normal physiological position, and finally improve the quality of life of the patient while improving the curative effect.

Restore cervical physiological radian was difficult to summarize clinical atlanto-axial abnormalities in patients with vertebral dislocation [11]. However, a new type of head and neck fixation and traction device could be used to achieve fixed traction before operation, which could reduce the atlantoaxial deformation, and thus lay a good foundation for the subsequent operation of reduction, so as to obtain a higher clinical traction effect [12]. A higher head and neck fixation effect could be obtained, so that the head of patients could maintain a normal physiological position, and finally improve the quality of life of patients while improving the curative effect [13].

## Conclusion

In conclusion, the posterior fusion treatment of patients with atlantoaxial joint dislocation with new head and neck deformity, assisted by nasal cannula oral release decompression fixation with fixed traction device, can enhance the fixation effect, help to increase the surgical efficacy of patients, and improve the quality of life of patients. It can improve the patient’s spinal cord function, effectively relieve the patient’s pain symptoms, and reduce the risk of surgery. It can promote the recovery of patients from many aspects and is worthy of clinical application. There are still some shortcomings in this study, such as relatively small sample size, short time span, incomplete results, etc., which may increase biased research data. Therefore, we should further expand the sample size in subsequent clinical studies. Based on the research of large samples and multiple centers, the results of this research are rich article end observation indicators, while improving the overall reference value of the research results.

## Data Availability

The datasets used and/or analyzed during the current study are available from the corresponding author on reasonable request.

## List of abbreviations

VAS: Visual Analogue Scale
ASIA: American Spinal Injury Association
JOA: Japanese Orthopeadic Association
QOLS: Quality of Life Scale
